# Relating Linear Energy Transfer to the Formation and Resolution of DNA Repair Foci After Irradiation with Equal Doses of X-ray Photons, Plateau, or Bragg-Peak Protons

**DOI:** 10.3390/ijms19123779

**Published:** 2018-11-28

**Authors:** Sebastian Oeck, Klaudia Szymonowicz, Gesa Wiel, Adam Krysztofiak, Jamil Lambert, Benjamin Koska, George Iliakis, Beate Timmermann, Verena Jendrossek

**Affiliations:** 1 Institute of Cell Biology (Cancer Research), University of Duisburg-Essen, Medical School, Virchowstrasse 173, 45122 Essen, Germany; klaudia.szymonowicz@uk-essen.de (K.S.); gesa.wiel@gmail.com (G.W.); adam.krysztofiak@uk-essen.de (A.K.); 2 Department of Therapeutic Radiology, Yale University School of Medicine, 15 York Street, New Haven, CT 06520, USA; 3 West German Proton Therapy Centre Essen, University Hospital Essen, Am Muehlenbach 1, 45147 Essen, Germany; jamil.lambert@uk-essen.de (J.L.); benjamin.koska@uk-essen.de (B.K.); beate.timmermann@uk-essen.de (B.T.); 4 Institute of Medical Radiation Biology; University of Duisburg-Essen; Medical School; Hufelandstr. 55, 45122 Essen, Germany; georg.iliakis@uk-essen.de

**Keywords:** Bragg-peak protons, plateau protons, photons, irradiation, DNA damage, foci formation, linear energy transfer

## Abstract

Proton beam therapy is increasingly applied for the treatment of human cancer, as it promises to reduce normal tissue damage. However, little is known about the relationship between linear energy transfer (LET), the type of DNA damage, and cellular repair mechanisms, particularly for cells irradiated with protons. We irradiated cultured cells delivering equal doses of X-ray photons, Bragg-peak protons, or plateau protons and used this set-up to quantitate initial DNA damage (mainly DNA double strand breaks (DSBs)), and to analyze kinetics of repair by detecting γH2A.X or 53BP1 using immunofluorescence. The results obtained validate the reliability of our set-up in delivering equal radiation doses under all conditions employed. Although the initial numbers of γH2A.X and 53BP1 foci scored were similar under the different irradiation conditions, it was notable that the maximum foci level was reached at 60 min after irradiation with Bragg-peak protons, as compared to 30 min for plateau protons and photons. Interestingly, Bragg-peak protons induced larger and irregularly shaped γH2A.X and 53BP1 foci. Additionally, the resolution of these foci was delayed. These results suggest that Bragg-peak protons induce DNA damage of increased complexity which is difficult to process by the cellular repair apparatus.

## 1. Introduction

Together with surgery and chemotherapy, radiotherapy is one of the three standard treatment options for cancer patients. At present, photon radiation is the most frequently used radiotherapy in the clinical setting. Photon radiation is an indirectly ionizing form of radiation (IR). Ionization is mediated mainly by the secondary electrons emitted by the photoelectric effect or the Compton effect [1,2]. In clinics, radiotherapy, with X-ray photons generated in a linear accelerator, has been the gold standard. A newer approach uses gamma photons from a Co-60 source and a focused radiation beam set-up [1,2,3]. Apart from radiotherapy with photons, proton therapy is increasingly used in cancer therapy as it may help to reduce normal tissue damage. Medically applied proton beams are usually produced in cyclotrons. In contrast to photons, protons and other charged particles can directly ionize atoms [2].

**Figure 1:**
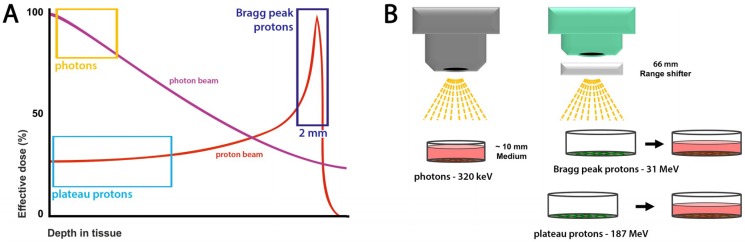
The relation between tissue depth and delivered dose of the different beams. (**A**) Depth dose curves of a photon beam and a proton beam highlighting the areas where the cells were irradiated with photons, Bragg-peak protons and plateau protons. (**B**) Experimental set-ups to simulate different tissue depths and radiation types for a photon beam, a Bragg-peak proton beam and a plateau proton beam. The cells were irradiated to a total dose of 3 Gy with each type of radiation calibrated using ionization chamber measurements at the same depth as the cells.

A key parameter for the quantification of the biological effects of radiation is the radiation dose; the energy in Joule delivered per Kg of irradiated material termed Gray (1 Gy = 1 J/kg). A second key parameter determining the biological effect of IR is the linear energy transfer (LET) (keV/µm) that describes the energy deposited per unit length of particle path [2,4,5]. Photon and proton beams differ fundamentally in their energy absorption profiles (Figure 1A): Photons lose their energy exponentially, with a higher value closer to the entry point. In contrast, protons deposit the majority of their energy only shortly before the particles come to a complete stop, the so-called Bragg-peak, as their mean energy loss per distance is inversely proportional to the square of their velocity, according to the Bethe–Bloch equation [4,5,6,7]. After the Bragg-peak, the delivered dose drops rapidly. As a consequence, high-energy protons can pass through normal tissue without losing a major portion of energy. Thus, the beams can deposit a much higher dose with higher precision deeper in the body where the tumor is located [4,8].

Despite the differences in the physical properties between photon and proton irradiation, the relative biological effectiveness (RBE) of these two radiation types is generally ascribed to a similar range, at least when compared to high LET particle beams, e.g., carbon ions [7,9,10,11]. The RBE depicts the ratio of the biologic effect between two different types of radiation measured per absorbed dose for a given endpoint (reference irradiation/test irradiation) to allow a comparison between two radiation types [4,12]. Clinical proton beam therapy mostly uses an RBE of 1.1 compared to the reference dose of gamma photons generated by a Co-60 source for clinical treatment planning. In contrast, published data point to variations of the value for proton RBE between 1.1 and 1.7, depending on the tissue, the measured end-point, and physical factors such as dose, fractionation, and LET of the beam, with potentially increasing values towards the end of the Bragg-peak [7,9,12].

In general, radiation that deposits a high amount of energy in a small area is more likely to induce DNA lesions with closer proximity to one another and such clustered damage sites may also be more difficult to be repaired [13,14,15,16,17]. In the present work, we aimed to gain insight into such potential biological differences between plateau and Bragg-peak protons. Therefore, we analyzed specific biologic endpoints related to the induction and processing of DNA double strand breaks (DSB). We exposed murine prostate cancer cells (TrC1) as well as murine embryonic fibroblasts (MEF) to irradiation with equal doses of X-ray photons, plateau protons and Bragg-peak protons and determined the kinetics of DNA damage induction and repair, mainly DSBs, by quantification of protein foci, phosphorylated Histone 2A member X (γH2A.X), and p53-binding protein 1 (53BP1). Moreover, we analyzed in detail the size, proximity, and form of the DNA repair foci. In this article, we define Bragg-peak protons as those with a residual range of less than 2 mm and plateau protons as those with a residual range of greater than 2 mm (Figure 1A).

Our results contribute to our understanding of the fluctuations of RBE of the protons and photons beams using as endpoints induction and processing of DNA DSB.

## 2. Results

### 2.1. Experimental Set-Ups for Equal Dose Irradiation with X-Ray Photons, Plateau Protons, and Bragg-Peak Protons

Due to the different physics of the beams, our first goal was to deliver an equal dose of 3 Gy to the cells at the dish surface. Therefore, we modified the set-up for the Bragg-peak proton, plateau proton, and photon irradiation accordingly. Figure 1A sketches the variation of the calculated effective dose of photon and proton beams traveling through tissue.

Our 320 keV photon beam had its delivered dose maximum at a depth of up to 1 cm in water. In our set-up, this tissue depth was simulated by 1 cm medium covering the cell monolayer. Here, an irradiation time of 47 s was necessary to achieve a dose of 3 Gy. Delivering a 3 Gy dose of Bragg-peak protons was more challenging; as energy deposition at the Bragg-peak occurs in a comparatively narrow area of tissue depth, it was crucial to simulate the tissue depth with accuracy in the millimeter range (Figure 1A). We achieved this by irradiating the cells with a transient complete removal of the culture medium during the irradiation procedure (Figure 1B). A Lexan range shifter, with a water equivalent thickness of 7.43 g/cm^2^, ensured an exact irradiation depth of the cell medium/tissue. A 105.5 MeV proton beam, decelerated by this range shifter to approximately 31 MeV, delivered 3 Gy to the cells within the Bragg-peak within a total irradiation time of 138 s. For the plateau proton set-up, we used a higher energy of 220 MeV to ensure that the proton beam was still in the plateau zone when reaching the cells after crossing the same range shifter. Consequently, the cells were hit by a proton beam with 187 MeV. These plateau protons traveled much faster through matter and deposited less energy on their track. Therefore, we had to increase the irradiation time to 234 s to achieve a dose of 3 Gy. Non-irradiated control cells were left for the same times without culture medium and outside the incubator. We didn’t take the differences of the irradiation duration into account, since these were comparably small to the time points after irradiation.

### 2.2. The Resolution of DNA Repair Foci is Delayed After Irradiation with Bragg-Peak Protons

**Figure 2:**
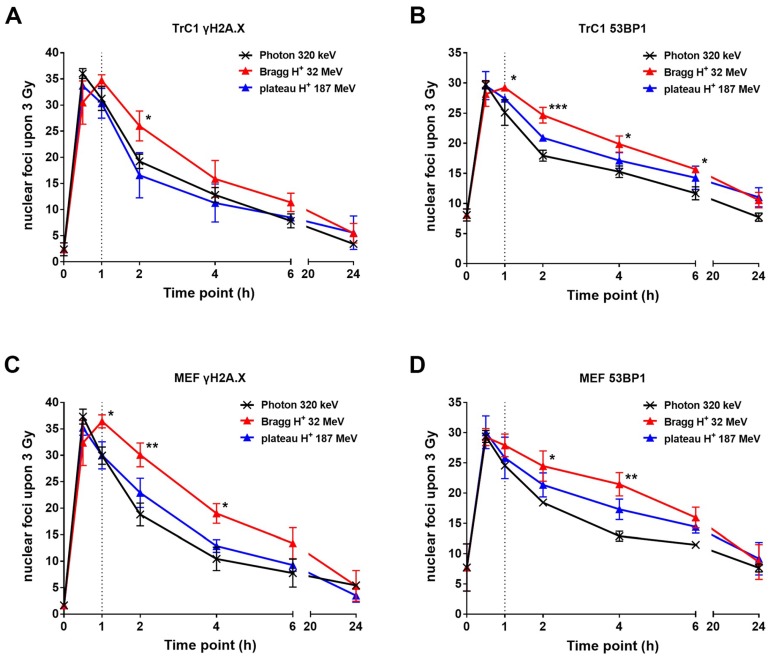
Formation and resolution of nuclear γH2A.X and 53BP1 foci after irradiation with X-ray photons or protons. Prostate cancer cells (TrC1) and murine embryonal fibroblasts (MEF) were exposed to 3 Gy irradiation with X-ray, plateau protons, and Bragg-peak protons. Cells were fixed at the indicated timepoints after irradiation for immunofluorescence analysis via γH2A.X (**A**,**C**) and 53BP1 (**B**,**D**). The γH2A.X and 53BP1 foci were analyzed with Focinator v2-22 software. The dotted line marks the 1-h timepoint. Data show means ± SD (*n* = 3, each 50 nuclei). * *p* < 0.05, ** *p* < 0.01, *** *p* < 0.001; multiple *t*-tests.

We performed different immunofluorescence-based assays to compare the amount of initial DSBs induced by treatment with 3 Gy of photons, plateau protons, or Bragg-peak protons, and to investigate the kinetics of DSB repair (Figure 2). For better visualization of single foci, we took mono-layer images generated with a Zeiss ApoTome and the Zen software. In contrast to the known foci counts in recent literature, the one-layer image, and thereby the removal of potential not-in-focus signals, resulted in a smaller foci count per nucleus. Furthermore, we used murine prostate cancer cells (TrC1) and murine embryonal fibroblasts (MEF), two adherent cell lines with large nuclear areas and no known alterations of the major DNA repair pathways, namely non-homologous end-joining (NHEJ) and homologous recombination repair (HRR). Monitoring the phosphorylation of γH2A.X and the accumulation of 53BP1 proteins at the sites of DNA damage is commonly used to visualize the amount of DSBs and the kinetics of their resolution as a consequence of time-dependent DSB repair. Thereby, it is possible to correlate foci persistence with DNA damage and repair. The phosphorylation of γH2A.X and the accumulation of 53BP1 is an early response that occurs within minutes after DNA damage and reaches maxima in foci count usually between 15 to 60 min after IR [18]. Both of our cell lines showed a maximum of about 35 γH2A.X foci (Figure 2A,C) and 30 53BP1 foci (Figure 2B,D) per nucleus 30 min after a dose of 3 Gy X-ray photons.

Further, we analyzed the kinetics of foci resolution over a time up to 24 h. At 2 h after IR, a considerable decrease in γH2A.X (Figure 2A,C) and 53BP1 (Figure 2B,D) foci could be observed in both cell lines, pointing to efficient DSB repair. Of note, the resolution of γH2A.X and 53BP1 foci was significantly slower after irradiation with Bragg-peak protons when compared to plateau protons and X-ray photons, at least at 2 h and 4 h after irradiation, suggesting a slower DSB repair. No significant differences could be detected in the kinetics of γH2A.X and 53BP1 foci resolution between plateau protons and X-ray photons. At 6 h after irradiation, the foci counts were comparable, regardless of cell line and type of radiation. Most of the foci resolved within 24 h with residual foci counts between 3 and 10 for γH2A.X and 10 and 15 for 53BP1, respectively.

Following this, we performed a more detailed analysis to examine whether different forms of IR alter foci size and distribution.

### 2.3. γH2A.X and 53BP1 Foci Differ in Size and Appearance after Different Types of Radiation

**Figure 3:**
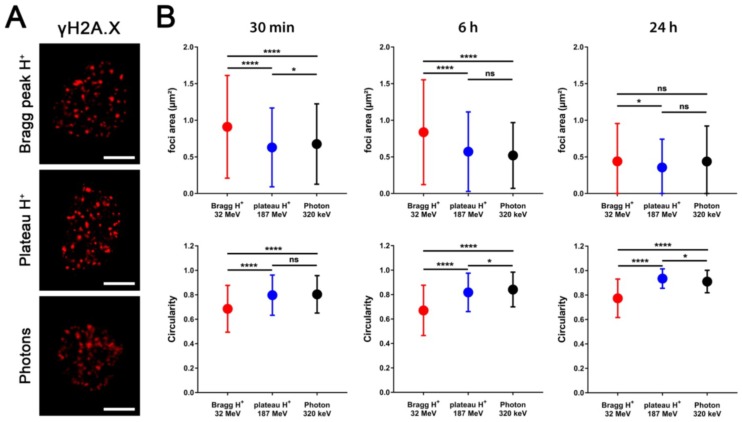

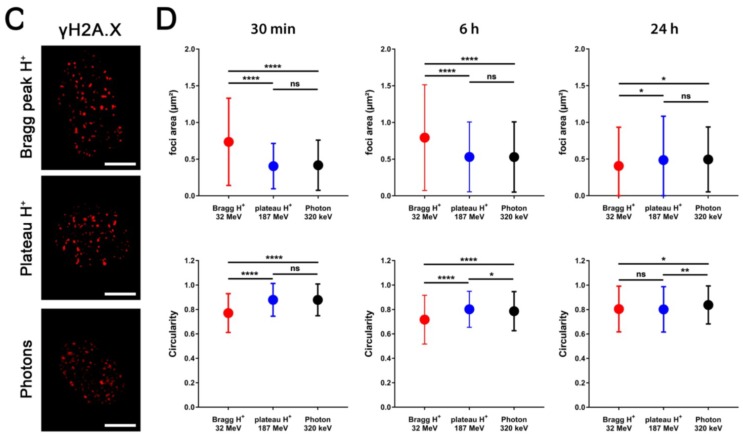
In-depth analysis of γH2A.X foci appearance from different types of IR. TrC1 (**A**,**B**) and MEFs (**C**,**D**) were fixed at distinct timepoints after 3 Gy of Bragg-peak proton, plateau proton, or photon irradiation, respectively. DSB sites were indirectly stained by γH2A.X immunofluorescence. (**A)** and (**C)** show representative high-resolution images (magnification 63×) of the 30 min timepoints, which were used for analysis of area, perimeter, and circularity of single foci (scale bar 5µm). The graph sets (**B**) and (**D**) display differences in foci area (upper panel) and circularity (lower panel) at three representative timepoints (30 min, 6 h, and 24 h) after different types of irradiation. Data represent mean values of at least 1000 foci/foci clusters ± SD obtained from three independent experiments. * *p* < 0.05, ** *p* < 0.01, **** *p* < 0.0001, ns = not significant; multiple *t*-tests.

Our data indicated maximal foci counts after IR with Bragg-peak protons only after 1 h, and thus later than after X-ray photon and plateau proton irradiation. To explore potential differences in foci morphology or localization, we additionally performed a detailed analysis of the size and distribution of these foci at different timepoints after IR (data shown for 30 min, 6 h, and 24 h after IR). We used high-resolution images (1388 x 1040 pixels, 63x) of TrC1 and MEF nuclei stained with γH2A.X (Figure 3A,C) and 53BP1 (Figure S2A,C) for the three different radiation types.

An ImageJ-based macro allowed us to analyze multiple parameters of hundreds of γH2A.X and 53BP1 foci. We chose three of these values to describe foci shape: The area in square micrometers (Figure 3B,D upper panels and Figure S2B,D upper panels), the perimeter of a single focus in micrometers (Figures S1 and S3), and the circularity, which is defined by $4\pi \times \mathrm{area}/{\mathrm{perimeter}}^{2}$ (Figure 3B,D lower panels, and Figure S2B,D lower panels). Irradiation with Bragg-peak protons led to extensive and bright γH2A.X and 53BP1 foci that seemed to be located in closer proximity to one another compared to plateau protons or X-ray photons at 30 min and 6 h after IR (Figure 3A,C, and Figure S2A,C). Moreover, the foci induced by Bragg-peak irradiation were characterized by a larger size compared to foci induced by plateau proton or X-ray photon irradiation (Figure 3B,D upper panels and Figure S2B,D upper panels). These differences in area size almost disappeared 24 h after IR and the residual foci were comparable in size and shape. Interestingly, we also observed diversity in circularity and perimeter of the foci after different radiation types. Considering that perfect circularity equals 1, our analysis revealed significantly altered circularity of γH2A.X and 53BP1 foci induced by Bragg-peak proton irradiation when compared to X-ray photons and plateau proton irradiation, respectively (Figure 3B,D lower panel and Figure S2B,D lower panel). Foci emerging after photon and plateau proton irradiation were almost perfectly circular after 24 h. Apart from a small difference in foci area 24 h after irradiation, plateau protons showed no significant differences compared to X-ray photons. In contrast, the Bragg-peak proton-induced foci appeared irregularly shaped with a significantly larger perimeter at all timepoints (Figures S1 and S3).

## 3. Discussion

In this paper, we introduced an innovative experimental set-up for irradiation of cell monolayers with an equal doses of single energy Bragg-peak protons and plateau protons. We aimed to explore variations in the biology of the induced DNA damage using 320 keV X-ray photons as reference irradiation. A direct comparison of the amount and appearance of γH2A.X and 53BP1 foci and of the kinetics of their resolution revealed small but significant spatiotemporal differences in the induction and/or processing of DSBs induced by single Bragg-peak proton beam compared to plateau protons and X-ray photons. The observed differences suggest that Bragg-peak protons can induce several DNA lesions in a restricted area potentially resulting in DNA lesions with higher complexity compared to plateau protons and X-ray photons. We could not detect significant differences between photons and plateau protons with respect to the analyzed parameters.

In more detail, irradiation with 3 Gy single Bragg-peak protons induced larger and more irregularly shaped γH2A.X and 53BP1 foci than irradiation with 3 Gy plateau protons or X-ray photons. For the latter, the induced γH2A.X and 53BP1 foci were smaller and had a more circular shape. Moreover, the maximum foci number upon irradiation with single Bragg-peak protons was only reached at 60 min post-irradiation compared to 30 min for plateau protons and photons and their resolution was delayed at 2 to 6 h post-irradiation. We speculate that the phenotype of larger and more irregularly shaped foci might be indicative of overlapping signals of two or three DNA lesions induced in closer proximity to one another than the smaller round foci observed upon irradiation with plateau protons or X-ray photons. During the progression of DNA repair, single smaller foci may become visible from the foci clusters and this might be one reason why the maximum number of foci is only observed 60 min upon irradiation with Bragg-peak protons. The induction of higher numbers of DNA lesions in closer proximity might also provide an explanation for the delay in the early repair of DNA damage induced by Bragg-peak protons compared to plateau protons and X-ray photons. As such, DNA lesions might be more difficult to repair. It has been proposed that irradiation with charged particles of higher LET, such as carbon ions induce highly clustered DNA lesions. This may be the cause for the slower repair observed [11,12,19].

So far, the concept of differences in the amount, the biology and/or the complexity of DNA lesions induced by Bragg-peak protons is still controversial. Furthermore, the dependency on specific DNA repair pathways needs to be investigated. Some authors have proposed that irradiation with clinically relevant Spread Out Bragg-peak protons (SOBP) is more effective in inducing DSBs compared to photon irradiation [20,21]. Other reports have suggested that the more pronounced toxic effects of protons in vitro and in vivo might be linked to their ability to cause more clustered, and thus more-difficult-to-repair, DSBs as a consequence of the higher energy transfer and the increased proximity of ionizing events, particularly for protons at the distal edge of the SOBP [4,10,13,17,22,23]. In contrast, other studies did not detect differences in DNA repair kinetics between photons and SOBP protons [24].

Potential differences in the RBE of protons with different LETs are also increasingly being studied in vivo. For example, recent reports correlated the use of protons from the distal edge of SOBP with significantly enhanced residual DSBs at 24 h after IR and increased radiosensitivity in esophageal cancer models [21,22]. Moreover, initial observations point to a potential biological effect of the increased LET of protons at the distal edge of the SOBP in normal tissue damage models in vivo [16,25]. It would be highly desirable to include comparisons between plateau protons, SOBP protons, and protons of the distal edge of the SOBP in such investigations.

We assume that such differences in the ability to induce higher numbers of DNA lesions in closer proximity between single Bragg-peak protons and SOBP protons, plateau protons, or photons might well contribute to the reported variations in the RBE between 1.1 and 1.7 for protons of certain energies, presumably by a higher ionization density per area [7,20]. Differences in the biology of DNA lesions between Bragg-peak protons and photons might also explain the reported dependency of cells exposed to SOBP protons on repair by homologous recombination repair (HRR) [26] as well as a higher diversity in histone post-translational modifications upon irradiation with Bragg-peak protons evoked as a consequence of more complex DNA damage [27]. Thus, understanding the mechanisms underlying the differences in the biology of irradiation with Bragg-peak protons, compared to plateau protons and photons and their consequences for radiosensitivity, might offer opportunities for proton therapy-specific strategies for targeted radiosensitization.

In conclusion, the differences in the cellular response to single Bragg-peak protons, compared to plateau protons and X-ray photons with respect to the formation and resolution of γH2A.X and 53BP1 foci, support earlier findings on differences in the biology of the DNA lesions induced by Bragg-peak protons and photons. Further studies could elucidate potential differences in cell survival in vitro and normal tissue toxicity in vivo. Moreover, this work suggests a potential direction for further studies revealing that such lesions might depend on a specific DNA repair pathway.

## 4. Materials and Methods

### 4.1. Chemicals, Antibodies and Drugs

Alexa Fluor 647-coupled antibody against γH2A.X protein was obtained from Becton Dickinson (Heidelberg, Germany). Anti-53BP1 rabbit polyclonal antibody was purchased from Bethyl Laboratories Inc. (Montgomery, TX, USA). Secondary antibodies Alexa Fluor 555 (anti-rabbit) and Hoechst33342 were purchased from Invitrogen (Eugene, OR, USA). DAKO Fluorescent mounting medium from Dako North America Inc. (Carpinteria, CA, USA) was used. All other chemicals were acquired from Sigma-Aldrich (Deisenhofen, Germany).

### 4.2. Cell Culture

TRAMP-C1 murine prostatic adenocarcinoma cells were purchased from ATCC (Bethesda, MD, USA). Murine embryonic fibroblasts were kindly provided by Morris J. Birnbaum (Philadelphia, PA, USA). Cells were cultured in DMEM (Thermo Fisher Technology, Waltham, MA, USA) medium supplemented with 10% (*v*/*v*) fetal calf serum (Biochrom AG, Berlin, Germany) and maintained in a humidified incubator (Labotect, Goettingen, Germany) at 37 °C and 5% CO_2_.

### 4.3. Irradiation

Cells were exposed to 3 Gy using different beams. Photon beams were produced with an X-RAD 320 X-ray Biological Irradiator with a MIR-324 X-ray tube (Precision X-Ray Inc., North Branford, CT, USA). Proton irradiation was performed on a Proteus Plus with a 230 MeV cyclotron (IBA International, Louvain-La-Neuve, Belgium). The irradiated fields covered a 300 × 300 mm single energy layer with pencil beam scanning and the cells in the isocenter. Bragg-peak proton irradiation was achieved by a 105.5 MeV proton beam travelling through a range shifter and almost no culture medium in the dishes. The range shifter was composed of 65 mm Lexan (1.14 g/cm^3^) and 1 mm water equivalent RW3 Slab Phantom (1.045 g/cm^3^) to adjust the range according to the field calibration (Sun Nuclear corp., FL, USA). Plateau proton irradiation was performed at 220 MeV through the same range shifter. Irradiation fields were calibrated by measuring the dose with a Dosimetry PPC05 parallel plate ionization chamber (IBA International, Louvain-La-Neuve, Belgium) at the same depth as the cells were during the irradiation.

### 4.4. Immunofluorescence Staining

Cells were fixed at distinct timepoints after irradiation. Non-irradiated controls were handled in parallel but kept outside of the irradiator during treatment to monitor putative effects not originating from the irradiation itself. Cells were fixed and permeabilized (3% paraformaldehyde (PFA) and 0.2% Triton X-100 in PBS; 15 min; RT). After washing with PBS, cells were blocked overnight with 2% goat serum in PBS. Antibodies were diluted in blocking buffer. Incubation with antibody against 53BP1 was performed for 1 h in a 1:500 dilution. Alexa Fluor 647-conjugated anti-γH2A.X antibody was incubated for 1 h at a 1:100 dilution. Staining with secondary antibody Alexa Fluor 555 (anti-rabbit) was performed for 1 h at a dilution 1:400. Samples were washed after each incubation step three times with PBS followed by staining for 15 min in the dark with 0.2% (*w*/*v*) Hoechst33342 in PBS. Samples were again washed with PBS, mounted with the DAKO mounting medium and stored at 4 °C in the dark. Single layer fluorescence images were taken with a Zeiss AxioCam MRm (1388 × 1040 pixels) at a Zeiss Axio Observer Z1 fluorescence microscope with Plan-Apochromat 63x/1.40 Oil M27 lens, 49 DAPI, 38 HE, 43 HE, and 78 HE ms filter and a transmission grid VH “ApoTome” (Carl Zeiss, Goettingen, Germany). Images were taken with three fourth of the maximum intensity without overexposure. The pictures were saved as 16-bit raw multi-channel Carl Zeiss Image files (CZI) with no further editing.

### 4.5. Software and Statistical Analysis

The Focinator v2 was used as previously described [18,28]. The software, instructions and supporting information are obtainable at http://www.focinator.com. Focinator v2 script was adapted to analyze the additional foci parameters. Data represent mean values of 3 independent experiments ± standard deviation (SD). Data analysis was performed by multiple *t*-tests or ANOVA and determination coefficient calculation using Prism6TM software (Graphpad Inc., La Jolla, CA, USA). *p* values ≤ 0.05 were considered as significant.

## Supplementary Materials

The Supplementary Materials are available online at http://www.mdpi.com/1422-0067/19/12/3779/s1.
