# Supplementary material for: Relating Linear Energy Transfer to the Formation and Resolution of DNA Repair Foci After Irradiation with Equal Doses of X-ray Photons, Plateau, or Bragg-Peak Protons
Source: Int J Mol Sci. 2018 Nov 28;19(12):3779. doi: 10.3390/ijms19123779 (PMC6320817; doi:10.3390/ijms19123779)
Supplement: Supplementary file 1 [file ijms-19-03779-s001.pdf]

International Journal of
Molecular Sciences

MDPI

Supplementary information

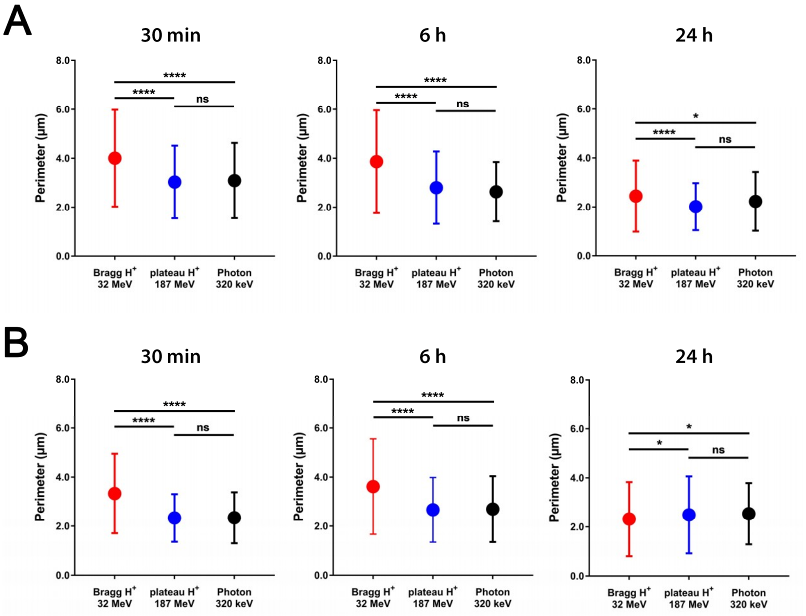

Figure S1. In-depth analysis of γH2A.X foci perimeter after different types of IR. Prostate cancer cells (TrC1) (**A**) and murine embryonic fibroblasts (MEFs) (**B**) were fixed at distinct timepoints after 3 Gy of Bragg-peak proton, plateau proton, or photon irradiation, respectively. The graph sets display differences in foci perimeter at three representative timepoints (0.5 h, 6 h, and 24 h) after different types of irradiation. Data represent mean values of at least 1000 foci ± SD obtained from three independent experiments. * $p < 0.05$, **** $p < 0.0001$, ns = not significant; multiple t-tests.

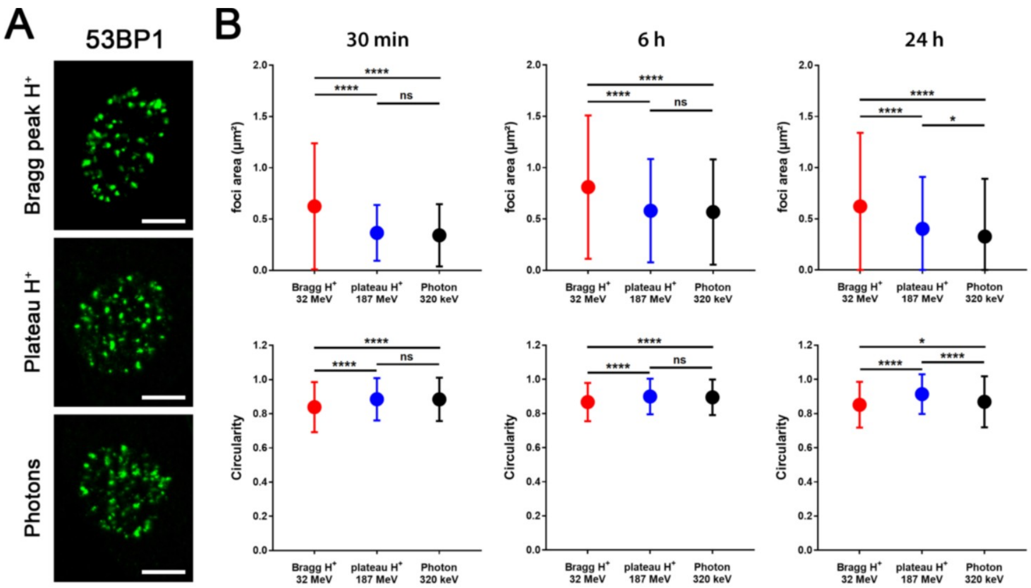

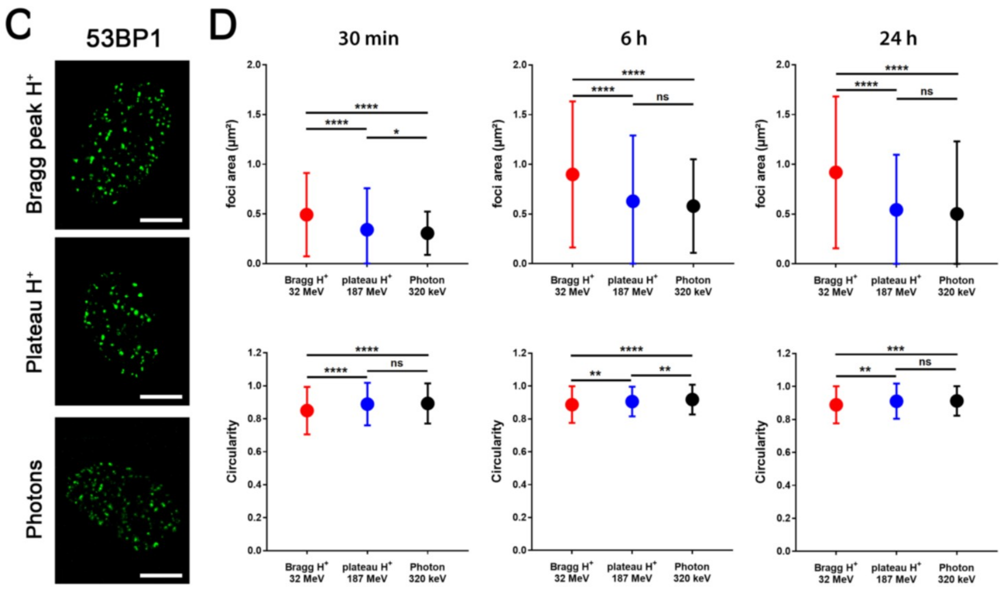

Figure S2. In-depth analysis of 53BP1 foci appearance after different types of IR. TrC1 (**A**, **B**) and MEFs (**C**, **D**) were fixed at distinct timepoints after 3 Gy of Bragg-peak proton, plateau proton, or X-ray photon irradiation, respectively. Double strand break (DSB) sites were indirectly stained for 53BP1 via immunofluorescence. **A** and **C** show representative high-resolution images which were used for analysis of area, perimeter, and circularity of single foci (scale bar 5 µm). The graph sets **B** and **D** display differences in foci area (upper panel) and circularity (lower panel) at three representative timepoints (0.5 h, 6 h, and 24 h) after different types of irradiation. Data represent mean values of at least 1000 foci ± SD obtained from three independent experiments. * $p < 0.05$, ** $p < 0.01$, *** $p < 0.005$, **** $p < 0.0001$, ns = not significant; multiple t-tests.

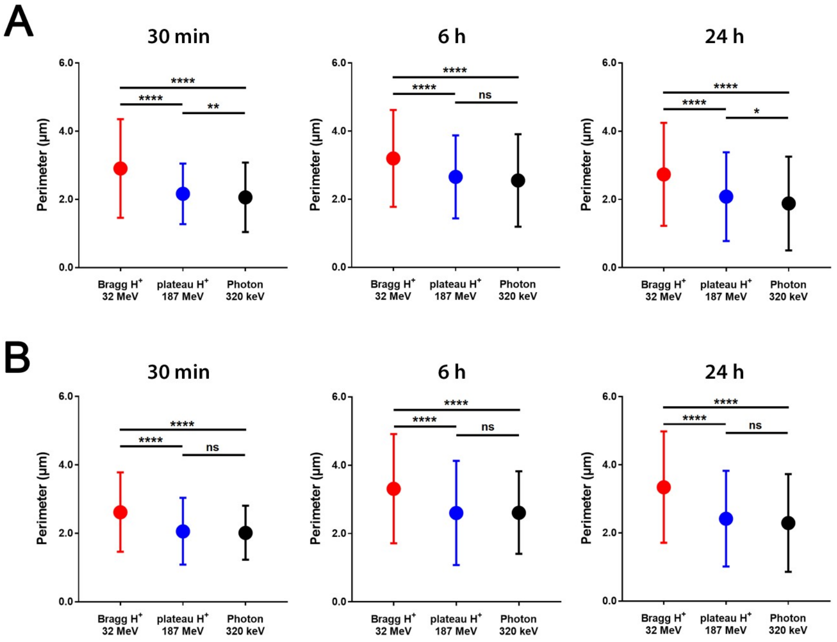

Figure S3. In-depth analysis of 53BP1 foci perimeter after different types of IR. TrC1 (**A**) and MEFs (**B**) were fixed at distinct timepoints after 3 Gy of Bragg-peak proton, plateau proton, or photon irradiation, respectively. The graph sets display differences in foci perimeter at three representative timepoints (0.5 h, 6 h, and 24 h) after different types of irradiation. Data represent mean values of at least 1000 foci ± SD obtained from three independent experiments. * $p < 0.05$, ** $p < 0.01$, **** $p < 0.0001$, ns = not significant; multiple t-tests.
